# Direct and highly productive conversion of cyanobacteria *Arthrospira platensis* to ethanol with CaCl_2_ addition

**DOI:** 10.1186/s13068-018-1050-y

**Published:** 2018-02-27

**Authors:** Shimpei Aikawa, Kentaro Inokuma, Satoshi Wakai, Kengo Sasaki, Chiaki Ogino, Jo-Shu Chang, Tomohisa Hasunuma, Akihiko Kondo

**Affiliations:** 1 Graduate School of Science, Technology, and Innovation, Kobe University, 1-1 Rokkodai, Nada-ku, Kobe, 657-8501 Japan; 2 Core Research for Evolutional Science and Technology, Japan Science and Technology Agency, 3-5 Sanbancho, Chiyoda-ku, Tokyo, 102-0075 Japan; 3 Graduate School of Engineering, Kobe University, 1-1 Rokkodai, Nada-ku, Kobe, 657-8501 Japan; 4 Department of Chemical Engineering, National Cheng Kung University, Tainan, 701 Taiwan; 5 Research Center for Energy Technology and Strategy, National Cheng Kung University, Tainan, 701 Taiwan; 6 Center for Bioscience and Biotechnology, National Cheng Kung University, Tainan, 701 Taiwan; 7 Biomass Engineering Program, RIKEN, 1-7-22 Suehiro, Tsurumi-ku, Yokohama, 230-0045 Japan; 8 Present Address: Biological Resources and Post-Harvest Division, Japan International Research Center for Agricultural Sciences (JIRCAS), 1-1 Ohwashi, Tsukuba, Ibaraki 305-8686 Japan

**Keywords:** Ethanol conversion, Glycogen extraction, Cyanobacteria, Amylase-displaying yeast, Polysaccharide layer, Organic nutrient

## Abstract

**Background:**

The cyanobacterium *Arthrospira platensis* shows promise as a carbohydrate feedstock for biofuel production. The glycogen accumulated in *A. platensis* can be extracted by lysozyme-degrading the peptidoglycan layer of the bacterial cell walls. The extracted glycogen can be converted to ethanol through hydrolysis by amylolytic enzymes and fermentation by the yeast *Saccharomyces cerevisiae*. Thus, in the presence of lysozyme, a recombinant yeast expressing α-amylase and glucoamylase can convert *A. platensis* directly to ethanol, which would simplify the procedure for ethanol production. However, the ethanol titer and productivity in this process are lower than in ethanol production from cyanobacteria and green algae in previous reports.

**Results:**

To increase the ethanol titer, a high concentration of *A. platensis* biomass was employed as the carbon source for the ethanol production using a recombinant amylase-expressing yeast. The addition of lysozyme to the fermentation medium increased the ethanol titer, but not the ethanol productivity. The addition of CaCl_2_ increased both the ethanol titer and productivity by causing the delamination of polysaccharide layer on the cell surface of *A. platensis*. In the presence of lysozyme and CaCl_2_, ethanol titer, yield, and productivity improved to 48 g L^−1^, 93% of theoretical yield, and 1.0 g L^−1^ h^−1^ from *A. platensis*, corresponding to 90 g L^−1^ of glycogen.

**Conclusions:**

We developed an ethanol conversion process using a recombinant amylase-expressing yeast from *A. platensis* with a high titer, yield, and productivity by adding both lysozyme and CaCl_2_. The direct and highly productive conversion process from *A. platensis* via yeast fermentation could be applied to multiple industrial bulk chemicals.

**Electronic supplementary material:**

The online version of this article (10.1186/s13068-018-1050-y) contains supplementary material, which is available to authorized users.

## Background

Cyanobacteria and green algae show promise as a carbohydrate feedstock for biorefinery and for the production of fuels and chemicals from biomass [1, 2]. They have a high carbohydrate content (> 50% of dry-cell weight) in nutrient-depleted conditions [3–5]. Cyanobacteria and microalgae, which primarily grow in aquatic environments, have the benefit of year-round cultivation using non-arable lands [6]. Some species, such as the cyanobacterium *Arthrospira platensis* or the green alga *Chlorella* sp., can convert solar energy into biomass more efficiently than energy crops such as switchgrass or C3 crops [7]. As summarized in Table 1, ethanol production from cyanobacteria and green algae by microbial fermentation has been developed in previous works.

**Table 1:** Ethanol production from cyanobacteria or green algae by microbial fermentation

Species	Hydrolysis procedure	Fermentation type	Ethanol titer (g L^−1^)	Ethanol yield (g-ethanol (g-DCW)^−1^)	Ethanol productivity (g L^−1^ h^−1^)	References
Green algae						
*Chlamydomonas reinhardtii*	Acid	SHF	15	0.29	0.61	[13]
*Chlorella* sp.	Acid	SHF	23	0.29	0.60	[14]
*Chlorella vulgaris*	Acid	SHF	12	0.23	1.2	[5]
*Scenedesmus obliquus*	Acid	SHF	12	0.02	1.1	[11]
*Scenedesmus acutus*	Acid	SHF	23	0.20	1.5	[15]
*C. reinhardtii*	Enzymatic	SHF	12	0.24	0.3	[10]
*C. vulgaris*	Enzymatic	SSF	4.3	0.21	0.16	[5]
Cyanobacteria						
*Synechococcus* sp.	Enzymatic	SHF	30	0.27	0.83	[12]
*A. platensis*	Enzymatic	SSF	6.0	0.32	0.60	[8]
*A. platensis*	Enzymatic	CBP	6.5	0.35	0.14	[8]
*A. platensis*	Enzymatic	CBP	48	0.32	1.0	This study

*SHF* separate hydrolysis and fermentation, *SSF* simultaneous saccharification and fermentation, *CBP* consolidated bioprocess, *DCW* dry-cell weight

The carbohydrates in cyanobacteria and green algae are mainly glucose polymers (polyglucans) such as starch, glycogen, or cellulose [5, 8, 9]. The *S. cerevisiae* and *Zymomonas mobilis* commonly used for ethanol production cannot hydrolyze polyglucans. Therefore, acid or enzymatic hydrolysis is commonly employed to obtain fermentable sugars, such as glucose, from cyanobacteria and green algae [5, 10–15, A1]. We have developed an ethanol production from the cyanobacterium *A. platensis* using a recombinant amylase-expressing yeast [8]. *A. platensis* accumulates glycogen (a glucose polymer linked linearly by α-1,4 glycosidic bonds with branches at the α-1,6 positions) intracellularly at 60–70% of dry-cell weight in nitrogen-depleted conditions [3]. Lysozyme, by degrading the peptidoglycan layer of bacterial cell walls, liberates the glycogen accumulated in *A. platensis* [8]. Amylolytic enzymes, including α-amylase and glucoamylase, can hydrolyze glycogen to glucose, which is fermentable by *S. cerevisiae*. Thus, in the presence of lysozyme, a recombinant *S. cerevisiae* strain expressing α-amylase and glucoamylase can produce ethanol directly from *A. platensis* with a high ethanol yield [0.35 g-ethanol (g dry-cell weight)^−1^] [8]. However, ethanol titer (6.5 g L^−1^) and ethanol productivity (0.14 g L^−1^ h^−1^) are much lower than those found in the previous research [8]. Achieving higher ethanol titers inevitably requires higher substrate loading during fermentation. Since ethanol productivity from the extracted glycogen of *A. platensis* is higher than that from non-pretreated *A. platensis* [8], we hypothesized that the rate of glycogen extraction from *A. platensis* limits ethanol productivity.

In the present study, to increase the ethanol titer, ethanol production was performed using a high concentration of *A. platensis* biomass. The polysaccharide layer on the cell surface of *A. platensis* includes lipopolysaccharide composing an outer membrane and extracellular polysaccharides [16], which form a barrier to glycogen extraction. To rapidly extract intercellular glycogen, we tried to enhance the permeability of the polysaccharide layer of *A. platensis*. In general, a high concentration of divalent cations such as Ca^2+^ or Mg^2+^ can increase the permeability of the polysaccharide layer of some bacteria, such as *Escherichia coli* or *Salmonella typhimurium* [17–19]. Pretreatment with 50 mM CaCl_2_, for instance, makes *E. coli* susceptible to DNA transfection and transformation [17]. Pretreatment of *S. typhimurium* cells with 100 mM MgCl_2_ enhances the release of periplasmic β-lactamase [18]. However, the effect of metal salts on the permeability of the polysaccharide layer of cyanobacteria has never been reported. We therefore examined the effect of metal salts on glycogen extraction from *A. platensis*. Conclusively, we succeeded in developing a direct and highly productive process for conversion of *A. platensis* to ethanol using a recombinant amylase-expressing yeast strain.

## Results and discussion

### Effects of lysozyme on ethanol production from a high concentration of *A. platensis* biomass

**Fig. 1:**
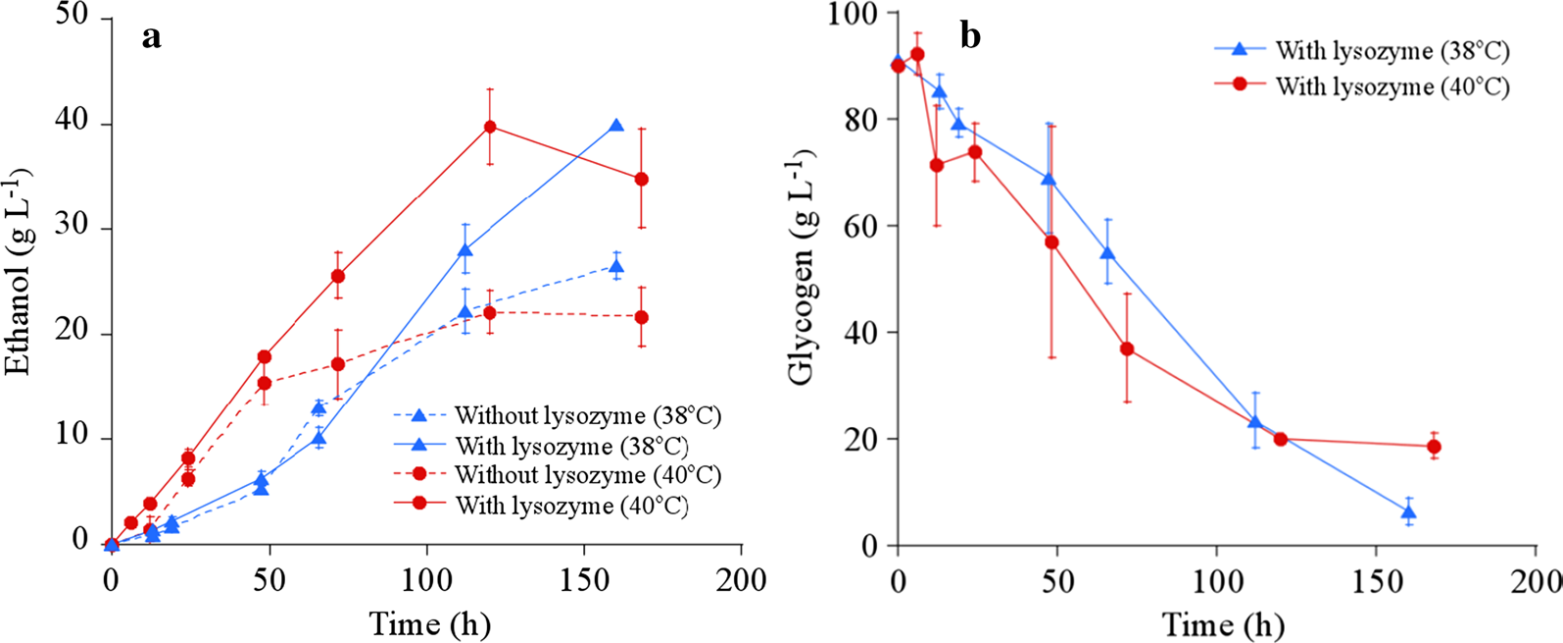
Ethanol production from a high concentration of *A. platensis* biomass in the presence of lysozyme. **a** Ethanol production in the presence of 1 g L^−1^ lysozyme (solid line) and the absence of lysozyme (dotted line) at 38 °C (blue triangle) and 40 °C (red circle). **b** Glycogen consumption in the presence of 1 g L^−1^ of lysozyme at 38 °C (blue triangle) and 40 °C (red circle). Error bars indicate standard deviations (SD) of three replicated experiments. For some data points, error bars obtained by three replications are smaller than the symbols

The addition of lysozyme, which degrades the peptidoglycan layer, to a fermentation medium enhances ethanol production from the dilute concentration of *A. platensis* biomass (20 g dry-cell weight L^−1^) [8]. We examined the effect of 1 g L^−1^ (6.7 mg (g dry-cell weight)^−1^) of lysozyme on ethanol production from a high concentration of *A. platensis* (150 g dry-cell weight L^−1^) using *S. cerevisiae* strain BY4741 AASS/GASS. The *Streptococcus bovis* α-amylase gene and *Rhizopus oryzae* glucoamylase gene are expressed by this strain. pH was maintained at 5.2–5.4 during yeast fermentation, without interference. Therefore, we did not control pH for yeast fermentation in this study. The ethanol production in the absence or presence of lysozyme at 38 and 40 °C is shown in Fig. 1a. In the absence of lysozyme, ethanol titer and ethanol yield were 26 g L^−1^ and 0.17 g-ethanol (g dry-cell weight)^−1^ at 38 °C, and 22 g L^−1^ and 0.15 g-ethanol (g dry-cell weight)^−1^ at 40 °C. The addition of lysozyme increased ethanol titer and ethanol yield to 40 g L^−1^ and 0.27 g-ethanol (g dry-cell weight)^−1^ at both 38 and 40 °C. These results indicate that lysozyme addition effectively enhances ethanol production from a higher concentration of *A. platensis* biomass. Concerning the effect of fermentation temperature on ethanol productivity in the presence of lysozyme, the ethanol productivity at 40 °C (0.33 g L^−1^ h^−1^) was superior to that at 38 °C (0.25 g L^−1^ h^−1^), as shown in Fig. 1a. Intracellular glycogen was released to the cell exterior, following lysis of *A. platensis* cells during fermentation. The total glycogen concentration of intracellular and extracted glycogens in the fermentation medium in the presence of lysozyme at 38 and 40 °C is shown in Fig. 1b. As shown in Fig. 1b, there was a tendency that the glycogen consumption at 40 °C was faster than that at 38 °C. This result agrees with the difference observed in the ethanol productivity in the presence of lysozyme between 38 and 40 °C shown in Fig. 1a. However, the ethanol productivity in the presence of lysozyme at 40 °C (0.33 g L^−1^ h^−1^) was still lower compared to that in the previous studies shown in Table 1 (0.60–1.5 g L^−1^ h^−1^). The degradation of the peptidoglycan layer by lysozyme would not sufficiently enhance ethanol productivity from *A. platensis*. Since the ethanol productivity from the extracted glycogen of *A. platensis* (0.60 g L^−1^ h^−1^) was faster than that from non-pretreated *A. platensis* cells (0.14 g L^−1^ h^−1^) as shown in Table 1 [8], we hypothesized that accelerating glycogen extraction from *A. platensis* during fermentation increases ethanol productivity.

### Effects of metal salts on polysaccharides on the cell surface of *A. platensis*

**Fig. 2:**
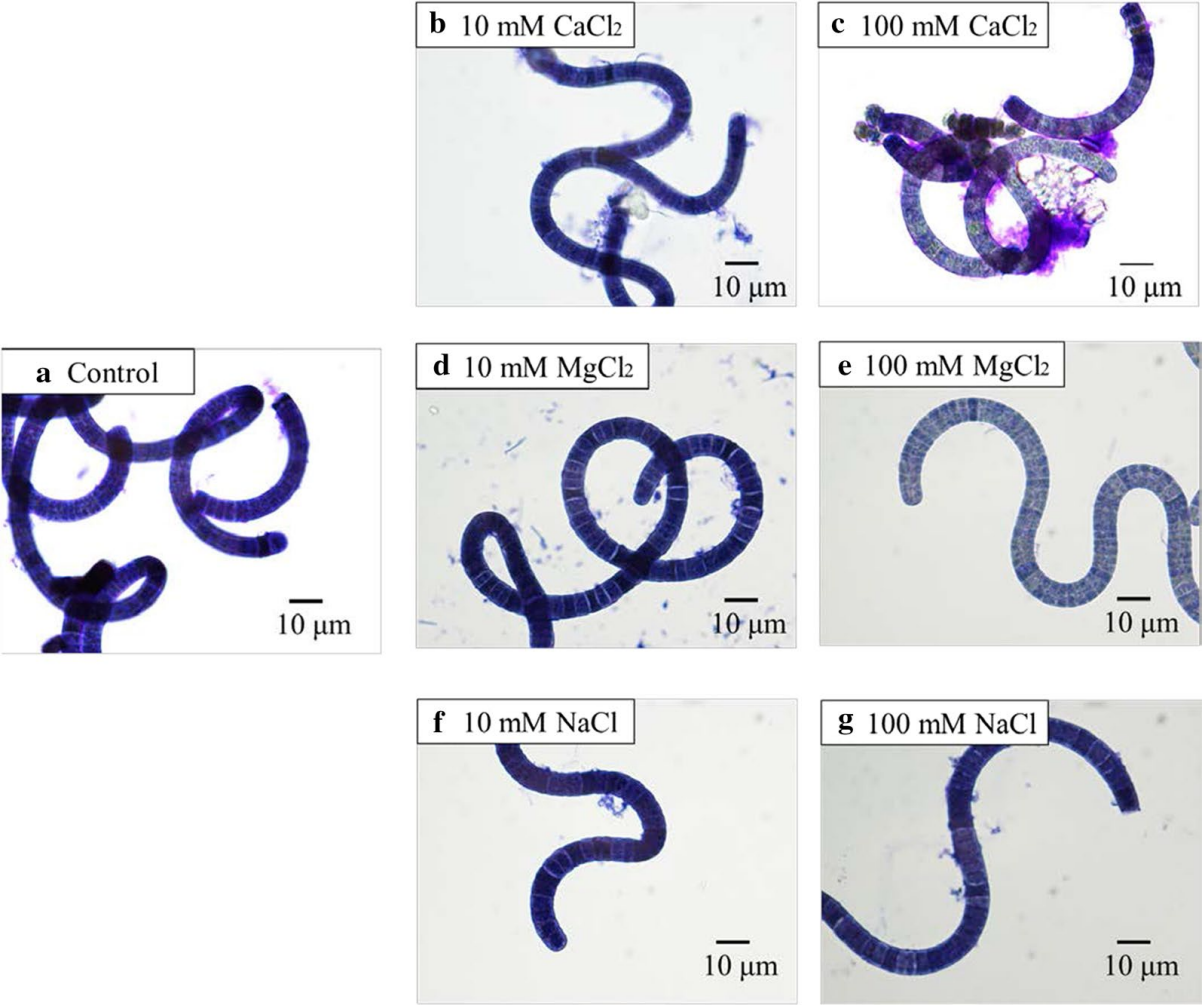
Effects of metal salts on polysaccharides on the cell surface of *A. platensis*. **a** Control (no addition of metal salts); **b** 10 mM CaCl_2_; **c** 100 mM CaCl_2_; **d** 10 mM MgCl_2_; **e** 100 mM MgCl_2_; **f** 10 mM NaCl; **g** 100 mM NaCl

We observed the effects of metal salts on the polysaccharide layer surrounding the *A. platensis* cells by staining with toluidine blue. Under control condition, the cell surfaces were uniformly stained purple with toluidine blue as shown in Fig. 2. The polysaccharide layer properly surrounded the *A. platensis* cells. In contrast, the cells treated with 100 mM CaCl_2_ were non-uniformly stained and aggregates, which are likely the delaminated polysaccharides, were observed around the cell surface. This phenomenon was not observed under the other tested conditions (10 mM CaCl_2_, 10 and 100 mM MgCl_2_, and 10 and 100 mM NaCl). Under these conditions, the cell surfaces were uniformly stained and aggregates were not observed as shown in Fig. 2. Multivalent metal cations, such as Ca^2+^, coagulate extracellular polysaccharides of a cyanobacterium *Microcystis aeruginosa* to form a gel [20]. Polysaccharide coagulation by a high concentration of Ca^2+^ would lead to delamination of the polysaccharides in 100 mM CaCl_2_. In contrast, Mg^2+^ and Na^+^ did not induce the polysaccharide delamination, regardless of the concentration. The valence of Na^+^ is insufficient for polysaccharide aggregation [20, 21]. The different structural changes in the polysaccharides induced by Ca^2+^ and Mg^2+^ have been previously reported [22–24]. The difference may be caused by differences in the hydration properties of Ca^2+^ and Mg^2+^, such as the Pauling radius, coordination number, or hydration energy [22, 23]. The cell surface polysaccharide in *A. platensis* has sulfate groups of unknown localization and carbohydrate groups of uronic acids, such as glucuronic acid and galacturonic acid [25]. Ca^2+^ would therefore preferentially bind to the sulfonate and carboxylate groups of the polysaccharides, as compared to Mg^2+^ [22]. Accordingly, the polysaccharide in *A. platensis* would be delaminated only at 100 mM CaCl_2_, as shown in Fig. 2.

### Effects of lysozyme and CaCl_2_ on glycogen extraction from *A. platensis*

**Fig. 3:**
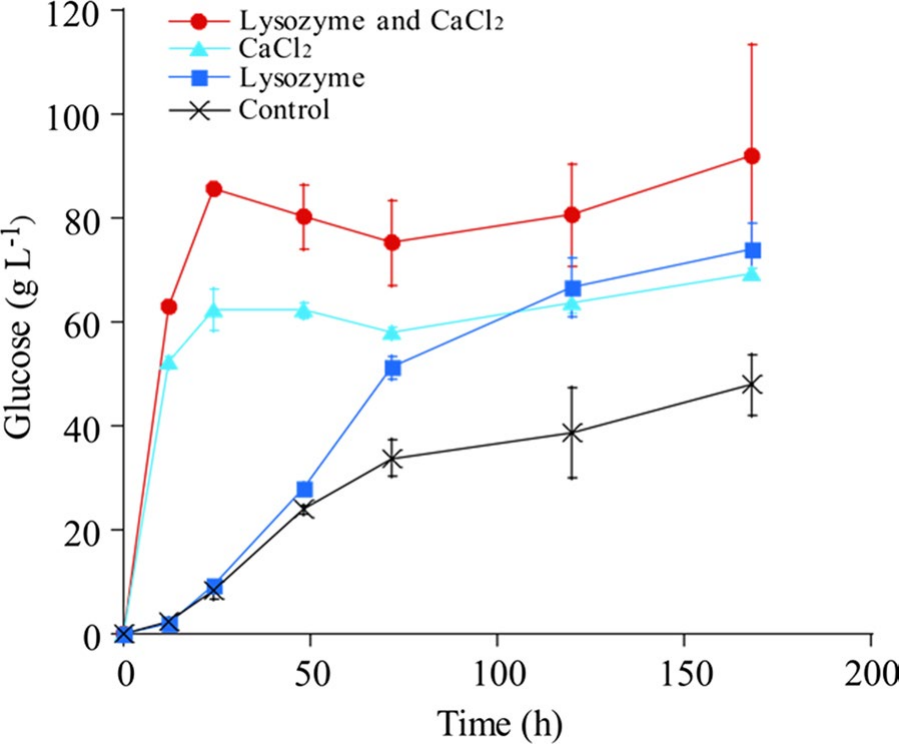
Effects of lysozyme and CaCl_2_ on glycogen extraction from *A. platensis*. Glucose concentrations in supernatant of the cell slurry of *A. platensis* in the presence of α-amylase and glucoamylase with neither lysozyme nor CaCl_2_ (black cross), lysozyme (blue square), CaCl_2_ (light blue triangle), and both lysozyme and CaCl_2_ (red circle) at 40 °C. Error bars indicate the SD of three replicated experiments. For some data points, error bars obtained by three replications are smaller than the symbols

We investigated the effects of lysozyme (1 g L^−1^) or CaCl_2_ (100 mM) on glycogen extraction from *A. platensis*, corresponding to 90 g L^−1^ of glycogen. This was carried out in 50-mL polypropylene tubes under axial rotation of 30 rpm at 40 °C. Only a limited amount of intracellular glycogen was extracted from *A. platensis* in the absence of carbohydrate-hydrolyzing enzymes. Therefore, glycogen was extracted in the presence of 0.3 U L^−1^ α-amylase and 0.1 U L^−1^ glucoamylase. The glucose concentration in the supernatant with neither lysozyme nor CaCl_2_, either lysozyme or CaCl_2_, and both lysozyme and CaCl_2_ is shown in Fig. 3. The glucose concentration was 43 g L^−1^ without lysozyme or CaCl_2_ after 168 h. Lysozyme addition to the cell slurry of *A. platensis* increased the glucose concentrations to 67 g L^‒1^ after 168 h. However, up to 48 h, the glucose concentration in the presence of lysozyme was similar to that without lysozyme or CaCl_2_ (Fig. 3). For instance, at 24 h, the glucose concentration was 9.4 g L^‒1^ with lysozyme and 8.3 g L^‒1^ without lysozyme or CaCl_2_. In contrast, the glucose concentration rapidly increased to 67 g L^−1^ with CaCl_2_ after 24 h (Fig. 3). Adding both lysozyme and CaCl_2_ further increased the glucose concentration to 83 g L^−1^ at 24 h. *A. platensis* cells would be disrupted more rapidly and thoroughly due to polysaccharide delamination on the cell surface by CaCl_2_ and degradation of the peptidoglycan layer by lysozyme, which would result in acceleration of glycogen extraction, as illustrated in Additional file 1.

### Ethanol production from *A. platensis* in the presence of lysozyme and CaCl_2_

**Fig. 4:**
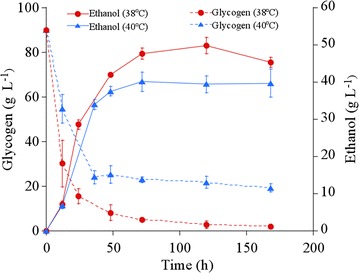
Ethanol production from *A. platensis* with CaCl_2_ addition in the presence of lysozyme. Ethanol production (solid line) and glycogen consumption (dotted line); ethanol was produced at 38 °C (blue triangle) and 40 °C (red circle). Error bars indicate SD of three replicated experiments. For some data points, error bars obtained by three replications are smaller than the symbols

We performed ethanol production from *A. platensis* by *S. cerevisiae* strain BY4741 AASS/GASS in the presence of both 1 g L^−1^ lysozyme and 100 mM CaCl_2_ (for enhancing glycogen extraction) at 38 and 40 °C. CaCl_2_ addition to the fermentation medium increased ethanol productivity to 1.0 g L^−1^ h^−1^ at both 38 and 40 °C (Fig. 4), although without addition the values were 0.25 g L^−1^ h^−1^ at 38 °C or 0.33 g L^−1^ h^−1^ at 40 °C (Fig. 1a). Here, *A. platensis* biomass was employed without physical pretreatment and enzymatic hydrolysis of carbohydrate. Nevertheless, the ethanol productivity was comparable to that from hydrolysates of cyanobacteria and green algae (0.60–1.5 g L^−1^ h^−1^; Table 1). These results support our hypothesis that accelerating glycogen extraction from *A. platensis* during fermentation can improve ethanol productivity. However, it is expected that ethanol productivity should be increased to > 1.5 g L^−1^ h^−1^, because the glucose concentration obtained from *A. platensis* was 83 g L^−1^ after 24 h (Fig. 3). The activity of amylases expressing on the yeast cell surface may limit ethanol productivity. To obtain higher ethanol productivity, higher amylase activity would be required. As shown in Fig. 4, CaCl_2_ addition increased the ethanol titer and yield at 38 °C to 48 g L^‒1^ and 0.32 g-ethanol (g dry-cell weight)^−1^. In contrast, the ethanol titer and the ethanol yield at 40 °C were 40 g L^−1^ and 0.27 g-ethanol (g dry-cell weight)^−1^, which were similar to those without CaCl_2_ at 40 °C (Fig. 1a). The glycogen consumption at 40 °C was slower than at 38 °C, and 20 g L^‒1^ of glycogen was left over at 40 °C (Fig. 4). Glucose was barely detectable in the supernatant at 40 °C during fermentation (data not shown). These results indicate that the activities of amylases displayed on the yeast cell surface were decreased at 40 °C. Retaining amylase activity would be vital to further improve the ethanol productivity in high-temperature conditions (> 40 °C). Finally, the addition of both CaCl_2_ and lysozyme improved the ethanol titer to 48 g L^−1^ at 38 °C. The ethanol titer obtained was larger than those found in the previous studies shown in Table 1. The energy required for ethanol distillation or membrane recovery and dehydration of ethanol is significantly higher when the ethanol titer is less than 40 g L^‒1^ [26–28]. Therefore, higher ethanol titers reduce the energy requirement of ethanol distillation.

### Ethanol production from *A. platensis* without organic nutrients by CaCl_2_ addition

**Fig. 5:**
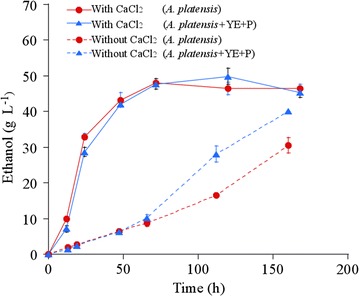
Ethanol production from *A. platensis* without organic nutrients, in the absence or presence of CaCl_2_. Ethanol production with organic nutrients (blue triangle) or without organic nutrients (red circle) at 38 °C in the presence of CaCl_2_ (solid line) and in the absence of CaCl_2_ (dotted line). YE and P mean yeast extract and bacto peptone, respectively. The data for ethanol production with organic nutrients and CaCl_2_ are the same as that in Fig. 4. The data for ethanol production with organic nutrients but without CaCl_2_ are same as that in Fig. 1a. Error bars indicate the SD of three replicated experiments. For some data points, error bars obtained by three replications are smaller than the symbols

Organic nutrients, such as yeast extract, peptone, or corn steep liquor, are generally necessary in a fermentation medium to support yeast fermentation [29]. The cost of organic nutrients is expensive for ethanol production. The lysates of the cyanobacteria *Synechococcus* sp., or the green algae *Scenedesmus acutus*, have a similar beneficial effect as organic nutrients during yeast fermentation [12, 15]. *A. platensis* contains nutrients such as free amino acids, vitamins, and minerals [30], which are likely utilized as a replacement of the organic nutrients in a fermentation medium. We carried out ethanol production from non-pretreated *A. platensis* by *S. cerevisiae* strain BY4741 AASS/GASS without organic nutrients in the presence of 1 g L^−1^ lysozyme and 100 mM CaCl_2_ at 38 °C, as shown in Fig. 5. The ethanol titer (48 g L^−1^), the ethanol yield [0.32 g-ethanol (g dry-cell weight)^−1^], and the ethanol productivity (1.0 g L^−1^ d^−1^) were similar to those obtained with organic nutrient addition. In contrast, in the presence of lysozyme and absence of CaCl_2_, the ethanol titer and productivity decreased when organic nutrients were not added (Fig. 5). The intracellular compounds efficiently extracted from *A. platensis* by lysozyme and CaCl_2_ would be supplied as nutrients for the yeast fermentation. These results indicate that direct ethanol production from *A. platensis* can be performed without exogenous nutrients for yeast fermentation.

### Advantages of ethanol production using an amylase-expressing yeast from *A. platensis*

The process of direct ethanol production from *A. platensis* used in this study has several advantages over conventional processes: (1) physical pretreatment and enzymes that can hydrolyze polyglucan are not required; (2) high titer, productivity, and yield of ethanol production; (3) pH neutralization prior to microbial fermentation is not required unlike processes that adopt chemical hydrolysis; (4) exogenous organic nutrients are not required during microbial fermentation. The entire procedure for ethanol production was simplified because the developed process combines polyglucan (glycogen) extraction, hydrolytic enzyme production, polyglucan hydrolysis, and yeast fermentation. The physical cell breakage for polyglucan extraction is commonly necessary for ethanol production with enzymatic hydrolysis from cyanobacteria and green algae [5, 12]. The addition of both 100 mM CaCl_2_ and 1 g L^−1^ of lysozyme can efficiently and rapidly extract glycogen from *A. platensis* without energy-consuming physical pretreatment. Ethanol production by a recombinant amylase-expressing yeast can omit amylase addition. *A. platensis* biomass was employed without physical pretreatment and polyglucan hydrolysis enzymes; nevertheless, the ethanol productivity and the ethanol yield were similar, and the ethanol titer was higher compared with the previous studies in Table 1. For ethanol production with chemical hydrolysis, H_2_SO_4_ or HCl was necessary for the polyglucan hydrolysis, and CaCO_3_ or NaOH was necessary for pH neutralization of hydrolysate [5, 11, 13, 14]. In contrast, the developed process did not require any chemicals for pH adjustment, which may contribute to a decrease in environmental load or energy consumption. Lysozyme addition is an issue in this process, which could be overcome by development of a recombinant lysozyme-expressing yeast. It is expected that this conversion process for *A. platensis* could be applied to the production of multiple industrial bulk chemicals, such as pyruvic acid or succinic acid, as well as ethanol [31].

For ethanol production, it is essential to reduce the costs related to the harvest and cultivation of feedstocks. The annual biomass production of cyanobacteria and green algae is 3–10 times higher than that of agricultural crops such as corn grain [6], but the costs of biomass production are 30–50 times more expensive [32, 33]. Cell harvesting of cyanobacteria and microalgae occupies 20–30% of the total cost of biomass production [32]. Although centrifugation can recover cyanobacterial and green-algal cells from the cultivation medium, centrifugation is too expensive for ethanol production. *A. platensis* cells, having a long spiral shape (20–100 μm length), can be harvested by the relatively cost-efficient and energy-efficient micro-screen harvesting method [34]. The cultivation of cyanobacteria and green algae for ethanol production is unlikely to be economically viable or provide a positive energy return without wastewater utilization [32]. *A. platensis* can be cultivated using various agro-industrial wastes and wastewater in the cultivation medium [35]. However, we have to adopt strategies to overcome the issues that inhibit cell growth due to contamination and decrease in light energy. The advantages of using *A. platensis* as feedstock would contribute to a reduction in the cost of bioethanol production.

## Conclusions

In the direct ethanol conversion process from *A. platensis* by a recombinant amylase-expressing yeast, the ethanol titer and productivity have been extremely low compared with the conventional process. We developed a direct ethanol conversion process from *A. platensis* with a high titer, yield, and productivity using both CaCl_2_ and lysozyme in the present study. The addition of lysozyme to degrade the peptidoglycan layer increased the ethanol titer. The removal of the polysaccharide layer on the cell surface by CaCl_2_ accelerated glycogen extraction from *A. platensis*, which markedly increased ethanol productivity. Our findings indicate that *A. platensis* is a valuable feedstock for ethanol production by microbial fermentation. To further improve the direct ethanol conversion process from *A. platensis*, a recombinant yeast that expresses lysozyme would be important. This direct conversion process of *A. platensis* by yeast fermentation could be further applied to the production of multiple industrial bulk chemicals, such as pyruvic acid or succinic acid.

## Methods

### Microorganism and growth conditions

A cyanobacterium *A. platensis* NIES-39 was obtained from the Global Environmental Forum (Tsukuba, Japan). *A. platensis* cells were pre-cultured in 500-mL Erlenmeyer flasks containing 250 mL of modified SOT medium with 100 rpm agitation under continuous illumination at 50-µmol photons m^−2^ s^−1^ for 7 days, with air-conditioning to maintain a temperature of 30 ± 2 °C in an NC350-HC plant chamber (Nippon Medical and Chemical Instruments, Osaka, Japan). The SOT medium consisted of 16.8 g L^−1^ NaHCO_3_, 0.5 g L^−1^ K_2_HPO_4_, 2.5 g L^−1^ NaNO_3_, 1.0 g L^−1^ K_2_SO_4_, 1.0 g L^−1^ NaCl, 0.2 g L^−1^ MgSO_4_·7H_2_O, 0.04 g L^−1^ CaCl_2_·2H_2_O, 0.01 g L^−1^ FeSO_4_·7H_2_O, 0.08 g L^−1^ Na_2_ EDTA, and 0.1% (v/v) A5 solution [30]. The A5 solution consisted of 2.86 g L^−1^ H_3_BO_3_, 2.5 g L^−1^ MnSO_4_·7H_2_O, 0.222 g L^−1^ ZnSO_4_·7H_2_O, 0.079 g L^−1^ CuSO_4_·5H_2_O, and 0.021 g L^−1^ Na_2_MoO_4_·2H_2_O. Pre-cultivated cells were inoculated at 0.03 (g dry-cell weight) L^−1^ and grown in 2-L flattened flasks containing 1.4 L SOT medium with 3 mM nitrate under continuous illumination at 500-µmol photons m^−2^ s^−1^ using white fluorescent bulbs (Life Look HGX and NHG; NEC, Tokyo, Japan) at 29 ± 1 °C with air bubbling at 350 mL min^−1^ for 3.5 days. The light intensity in the center of the medium was measured using an LI-250A light meter (LI-COR, Lincoln, NE) equipped with an LI-190SA quantum sensor (LI-COR).

*Saccharomyces cerevisiae* strain BY4741 AASS/GASS, which expressed the α-amylase gene from *Streptococcus bovis* and glucoamylase gene from *Rhizopus oryzae* [36], was grown aerobically in 500 mL YPD medium (10 g L^−1^ yeast extract, 20 g L^−1^ peptone, and 20 g L^−1^ glucose) at 30 °C with agitation at 150 rpm for 2 days prior to fermentation.

### Observations of polysaccharides on the cell surface of *A. platensis*

*Arthrospira platensis* cells cultivated for 7 days in SOT medium with 30 mM NaNO_3_ were collected with centrifugation (6300×*g* for 2 min at 25 °C), and then washed once with distilled water. The *A. platensis* cells were transferred at 0.7 (g dry-cell weight) L^−1^ to 10 mL of distilled water without additives, and 10 mM CaCl_2_, 100 mM CaCl_2_, 10 mM MgCl_2_, 100 mM MgCl_2_, 10 mM NaCl, or 100 mM NaCl were added, each to one of 6-well plates. The mixtures were then rotated at 100 rpm in an incubator HB-80 (TAITEC, Tokyo, Japan) for 30 min at 40 °C. The lipopolysaccharide of *A. platensis* for each condition was stained by 0.05% (w/v) toluidine blue (Nacalai Tesque, Kyoto, Japan) [37]. The polysaccharides on the cell surface of *A. platensis* were observed with a light microscope Eclipse TE 300 (Nikon, Tokyo, Japan), and images were digitized using a digital camera DS-Ri1 (Nikon).

### Glycogen extraction and ethanol fermentation from *A. platensis*

*Arthrospira platensis* cells, cultivated for 3.5 days in SOT medium with 3 mM nitrate, were collected with a nylon net filter (30 cm × 40 cm, 20-µm pore size; Millipore, Billerica, MA), and then washed once with distilled water while still on the filter. To achieve high-titer ethanol production, a high-biomass concentration (1.5 kg wet-cell weight L^−1^) of *A. platensis* was used as the carbon source for yeast fermentation. The biomass volume was reduced by removing 40% (w/w) of the raw biomass water content with cellulose absorptive sponges (15 cm × 9 cm × 3 cm, Toray Fine Chemicals, Chiba, Japan) and dehydrated by pressing at 500 g × 100 cm^−2^. High-yield ethanol production from high-solid lignocellulosic biomass was performed in a drum-type rotatory fermenter to achieve sufficient mixing of the high-viscosity biomass [38]. Due to its simplicity and high potential for large-scale fermentation [39], a drum-type rotary fermenter (Thermo Block Rotator SN-06BN; Nissin, Tokyo, Japan), which axially rotating 50-mL polypropylene tubes (Corning Inc., NY) at 30 rpm, was used for ethanol production from *A. platensis* biomass in the present study. The vessel was sealed with a silicon plug, into which a hole was bored using a disposable needle (φ = 0.6 mm) (Terumo Corp., Tokyo, Japan). Yeast cells were collected by centrifugation at 3000×*g* for 3 min at 25 °C, washed twice with distilled water, and inoculated with 50 (g wet-cell weight) L^−1^ into YP medium (10 g L^−1^ yeast extract and 20 g L^−1^ peptone) or distilled water containing *A. platensis* cells corresponding to 90 g L^−1^ glycogen. For the extraction of intracellular glycogen from *A. platensis*, 1 g L^−1^ lysozyme and 100 mM CaCl_2_ were added to the fermentation medium. Ethanol production with the *S. cerevisiae* strain BY4741 can be performed at 38–40 °C [40]. High-temperature fermentation offers advantages such as reduced contamination risks and cooling costs, and is therefore suitable for tropical countries [41]. Therefore, we produced ethanol from *A. platensis* at 38 and 40 °C using the *S. cerevisiae* strain BY4741 AASS/GASS.

### Analytical methods

The ethanol concentrations were analyzed using a GC2010 Plus gas chromatograph (Shimadzu, Kyoto, Japan) equipped with a GC-FID flame ionization detector (Shimadzu) and a DB-FFAP column (60 m × 0.250 mm i.d., 0.5 µm film thickness; Agilent, Palo Alto, CA) with helium as the carrier gas. The column temperature was held at 40 °C for 1 min before being raised to 170 °C with a linear gradient of 10 °C min^−1^. The injector and detector temperatures were maintained at 230 °C. The injection volume was 1 μL and the split ratio was adjusted to 1:50.

The glycogen content of *A. platensis* cells was determined by HPLC (Shimadzu, Kyoto, Japan) using a size-exclusion HPLC column (OHpak SB-806M HQ; Shodex, Tokyo, Japan) and a reflective index detector (RID-10A; Shimadzu, Kyoto, Japan), as previously described [42]. The concentration of extracted glycogen from *A. platensis* was calculated from the glucose concentration. The glucose concentration was determined using a high-performance liquid chromatograph (HPLC) (LC20A; Shimadzu) with an ion-exchange HPLC column (Unison UK-Amino UKA06; Imtakt, Kyoto, Japan) and an evaporative light scattering detector (ELSD-LTII; Shimadzu). Experimental data are shown as the triplicate sample means and error bars indicate the standard deviation.

The α-amylase and glucoamylase activities were measured using an α-amylase assay kit and a saccharifying ability assay kit (Kikkoman Corp., Chiba, Japan), respectively; 2-chloro-4-nitrophenyl 6^5^-azide-6^5^-deoxy-β-maltopentaoside and 4-nitrophenyl-β-d-maltoside were used as the substrates according to the manufacture’s instruction.

## Additional file

**Additional file 1.** Schematic diagram of glycogen extraction from *A. platensis* in the presence of lysozyme and CaCl_2_.

## Abbreviations

CBP: consolidated bioprocess
HPLC: high-performance liquid chromatograph
SHF: separate hydrolysis and fermentation
SSF: simultaneous saccharification and fermentation
