# Supplementary figures and images for: Direct and highly productive conversion of cyanobacteria Arthrospira platensis to ethanol with CaCl2 addition
Source: Biotechnol Biofuels. 2018 Feb 27;11:50. doi: 10.1186/s13068-018-1050-y (PMC5828149; doi:10.1186/s13068-018-1050-y)

## Slide 1
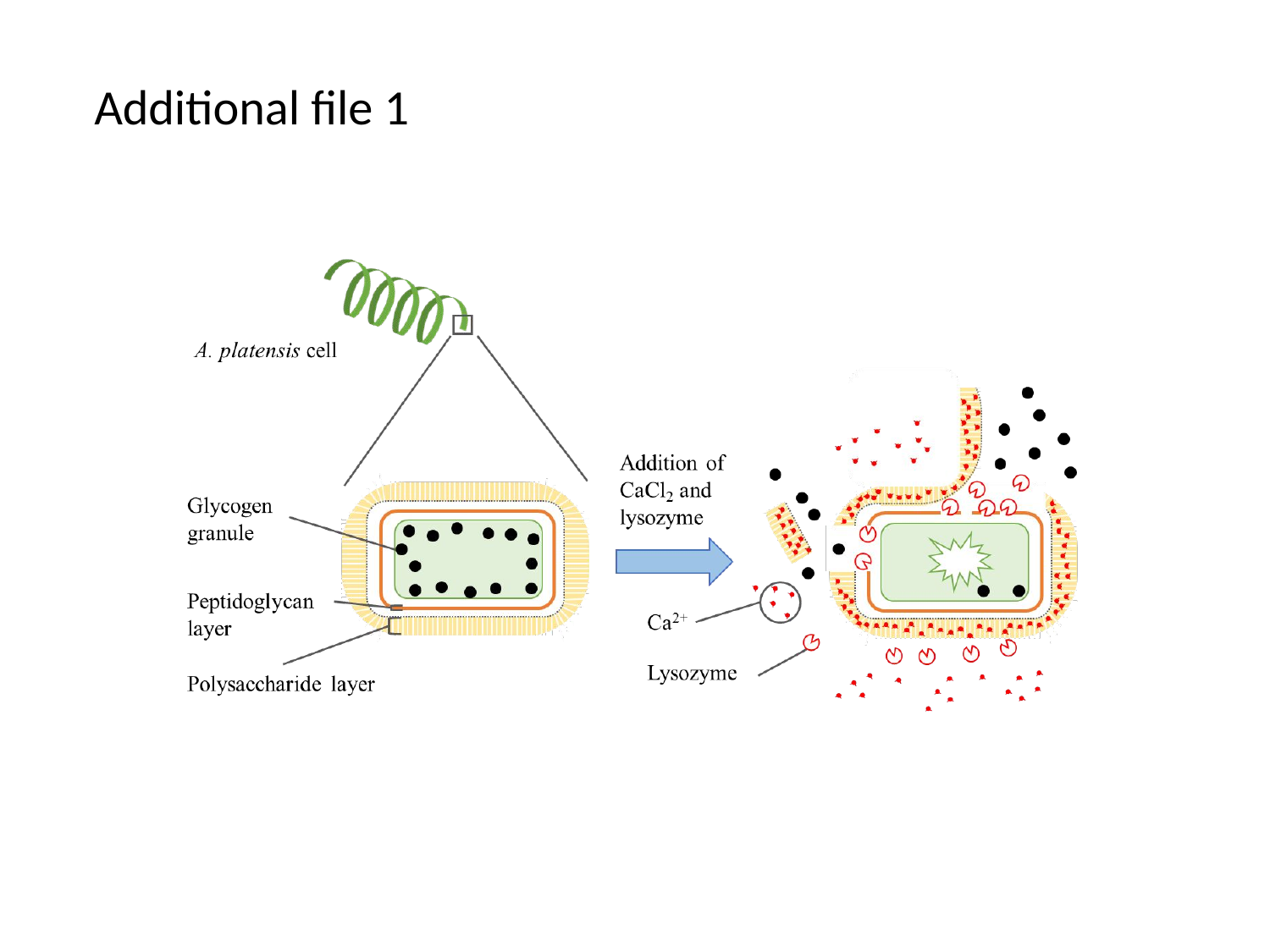

Additional file 1

Supplement: Supplementary file 1 — Additional file 1. Schematic diagram of glycogen extraction from A. platensis in the presence of lysozyme and CaCl2. [file 13068_2018_1050_MOESM1_ESM.pptx]
